# Efficient and Non-genotoxic RNA-Based Engineering of Human T Cells Using Tumor-Specific T Cell Receptors With Minimal TCR Mispairing

**DOI:** 10.3389/fimmu.2018.02503

**Published:** 2018-11-07

**Authors:** Diana Campillo-Davo, Fumihiro Fujiki, Johan M. J. Van den Bergh, Hans De Reu, Evelien L. J. M. Smits, Herman Goossens, Haruo Sugiyama, Eva Lion, Zwi N. Berneman, Viggo Van Tendeloo

**Affiliations:** ^1^Faculty of Medicine and Health Sciences, Vaccine & Infectious Disease Institute (VAXINFECTIO), University of Antwerp, Antwerp, Belgium; ^2^Department of Cancer Immunology, Osaka University Graduate School of Medicine, Osaka, Japan; ^3^Center for Cell Therapy & Regenerative Medicine, Antwerp University Hospital, Edegem, Belgium; ^4^Faculty of Medicine and Health Sciences, Center for Oncological Research (CORE), University of Antwerp, Antwerp, Belgium; ^5^Division of Clinical Biology, Antwerp University Hospital, Edegem, Belgium; ^6^Division of Hematology, Antwerp University Hospital, Edegem, Belgium

**Keywords:** TCR-gene transfer, electroporation, adoptive cell therapy (ACT), DsiRNA, RNA transfection

## Abstract

Genetic engineering of T cells with tumor specific T-cell receptors (TCR) is a promising strategy to redirect their specificity against cancer cells in adoptive T cell therapy protocols. Most studies are exploiting integrating retro- or lentiviral vectors to permanently introduce the therapeutic TCR, which can pose serious safety issues when treatment-related toxicities would occur. Therefore, we developed a versatile, non-genotoxic transfection method for human unstimulated CD8^+^ T cells. We describe an optimized double sequential electroporation platform whereby Dicer-substrate small interfering RNAs (DsiRNA) are first introduced to suppress endogenous TCR α and β expression, followed by electroporation with DsiRNA-resistant tumor-specific *TCR* mRNA. We demonstrate that double sequential electroporation of human primary unstimulated T cells with DsiRNA and *TCR* mRNA leads to unprecedented levels of transgene TCR expression due to a strongly reduced degree of TCR mispairing. Importantly, superior transgenic TCR expression boosts epitope-specific CD8^+^ T cell activation and killing activity. Altogether, DsiRNA and *TCR* mRNA double sequential electroporation is a rapid, non-integrating and highly efficient approach with an enhanced biosafety profile to engineer T cells with antigen-specific TCRs for use in early phase clinical trials.

## Introduction

Cancer is one of the leading causes of death in the world, according to the World Health Organization. Traditionally, the first lines of cancer treatment are chemotherapy, radiotherapy and/or surgery. However, the high incidence of relapse among cancer patients led to the development of new strategies exploring the use of our immune system as a refined and more specific tool to fight cancer (1). In particular, among the different cancer immunotherapies available, adoptive cell transfer of T cells has been the focus of numerous advances in medicine. In fact, the potential of adoptive T-cell therapy has been demonstrated in both malignant and infectious diseases (2). In cancer immunotherapy, many of these therapies focus on tumor associated antigens (TAAs) that are overexpressed in cancer cells and are only present in limited amounts in other healthy tissues (3). Yet the negative selection of self-antigen reactive T cells translates into scarcity of circulating TAA-specific T cells, challenging their *ex vivo* isolation and demanding timely and large-scale *ex vivo* expansion (4). To circumvent this limitation, T cell receptor (TCR) gene engineering of bulk T cells is increasingly becoming the method of choice to produce large amounts of redirected T cells (5). However, the clinical efficacy of TCR-redirected T cells is still not satisfactory, and serious adverse effects have been observed in clinical trials (5). First, gene transfer methods involving transduction by retro- or lentiviral vectors can integrate viral DNA into the host genome potentially leading to insertional mutagenesis that could disrupt genes important for cell function or promote tumorigenesis (6). Second, in the event of unanticipated transgenic TCR specificities, permanent expression of transgenic TCR could produce long-lasting toxicities with severe consequences (5, 6). Third, strategies to improve the efficacy of the therapy, including modifications of transgenic TCR structure via introduction of murine domains to enhance preferential pairing or artificial enhancement of TCR affinity could result in undesired immunogenicity, are technically demanding and costly (7, 8). Fourth, concomitant expression of endogenous and transgenic *TCR* genes produces two sets of TCR alpha (TCRα) and beta (TCRβ) chains that can pair incorrectly (9), generating two mispaired TCR heterodimers that reduce transgenic TCR levels (10) and may lead to on-target and off-target toxicities in patients (11). These data have prompted us to develop a safer, faster and more widely applicable method for TCR engineering of T cells. Based on our longstanding expertise with clinical tools using mRNA-modified dendritic cell (DC) vaccines in acute myeloid leukemia (AML) patients (12, 13), we adapted our mRNA electroporation protocol to human resting CD8^+^ T cells for rapid and efficient transient TCR expression (14, 15, 16, 17). Furthermore, we implemented an RNA interference step for substantial reduction of TCR mispairing, enhancing the safety profile of TCR-engineered T cells. Overall, we present a double sequential electroporation of DsiRNA and codon-optimized *TCR* mRNA as a non-genotoxic, highly efficient and versatile non-viral platform with an enhanced biosafety profile to engineer T cells with TCRs for adoptive T cell immunotherapy.

## Results

### Cloning of WT1-specific *TCR* mRNA and validation in a 2D3 cell model

**Figure 1:**
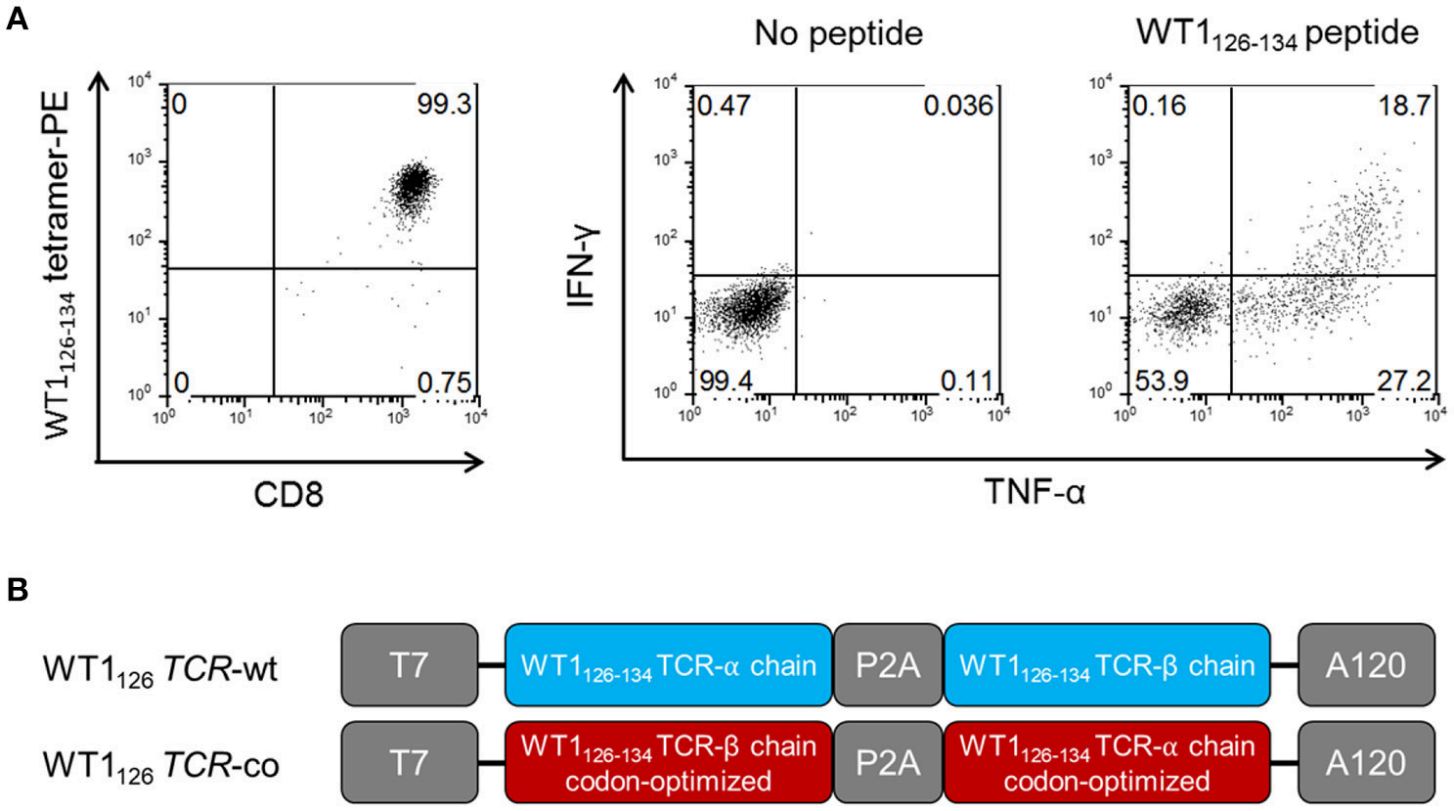
Isolation and characterization of WT1_126_-specific CTL clone. **(A)** WT1_126−134_/HLA-A*02:01 tetramer staining and WT1_126−134_ peptide-specific interferon (IFN)-γ and tumor necrosis factor (TNF)-α production of the WT1_126−134_-reactive CTL clone. **(B)** Schematic representation of pST1 plasmid vectors containing the WT1_126−134_-specific wild-type (WT1_126_ *TCR*-wt) and WT1_126−134_-specific codon-optimized (WT1_126_ *TCR*-co) TCR cassettes. WT1, Wilms' tumor 1; wt, wild-type; co, codon-optimized; T7, T7 promoter; P2A, picornaviral 2A-like sequence; A120, 120-mer poly-A tail.

We established a cytotoxic T lymphocyte (CTL) clone reactive to WT1_126−134_ peptide from an HLA-A^*^02:01^+^ patient with acute myeloid leukemia (AML) with a favorable clinical response in our Wilms' tumor protein 1 (WT1)-targeted DC vaccination trial (ClinicalTrials.gov NCT00834002) and with polyepitope WT1-specific CTL responses (12) (Figure 1A). After isolation of *TCR*α and *TCR*β genes, the wild-type *TCR*α and *TCR*β sequences were linked with a P2A peptide sequence (18) and inserted into a plasmid vector for bicistronic and equimolar expression of both TCR chains (WT1_126_ *TCR*-wt mRNA; Figure 1B). To enhance *TCR* mRNA translation, the *TCR*α and *TCR*β sequences were codon-optimized and the order of the *TCR* genes was reversed (19), inserting the *TCR*β before the P2A peptide sequence (WT1_126_ *TCR*-co mRNA; Figure 1B). After *in vitro TCR* mRNA generation, we validated transgenic TCR expression in a 2D3 cell line originating from TCRαβ-deficient Jurkat 76 cells (Figure S1). High levels of WT1_126_ TCR were detected in 2D3 cells 4 hours (h) after WT1_126_ *TCR*-wt or WT1_126_ *TCR*-co mRNA electroporation (56.3 ± 0.3% and 71.9 ± 1.5%, respectively; mean ± SEM of 3 replicates). WT1_126_ TCR expression was higher after transfection with *TCR*-co mRNA as compared to *TCR*-wt mRNA at most time points post-electroporation, whilst transgenic TCR was lost 5 days after transfection with either mRNA (Figure S1A). To analyze the functional avidity of the cloned TCR, WT1_126_ *TCR*-wt or WT1_126_ *TCR*-co mRNA-electroporated 2D3 cells were cultured with T2 cells pulsed with titrated amounts of WT1_126−134_ peptide. TCR functionality was confirmed by marked expression levels of enhanced green fluorescent protein (*EGFP*) reporter gene at high peptide concentrations for both *TCR*-wt and *TCR*-co mRNA-electroporated 2D3 cells, with identical TCR activation thresholds at a WT1_126−134_ peptide concentration of 10^−3^ μM (Figure S1B).

### Electroporation of DsiRNA targeting *TRAC* and *TRBC* transcripts inhibits endogenous TCR expression

**Figure 2:**
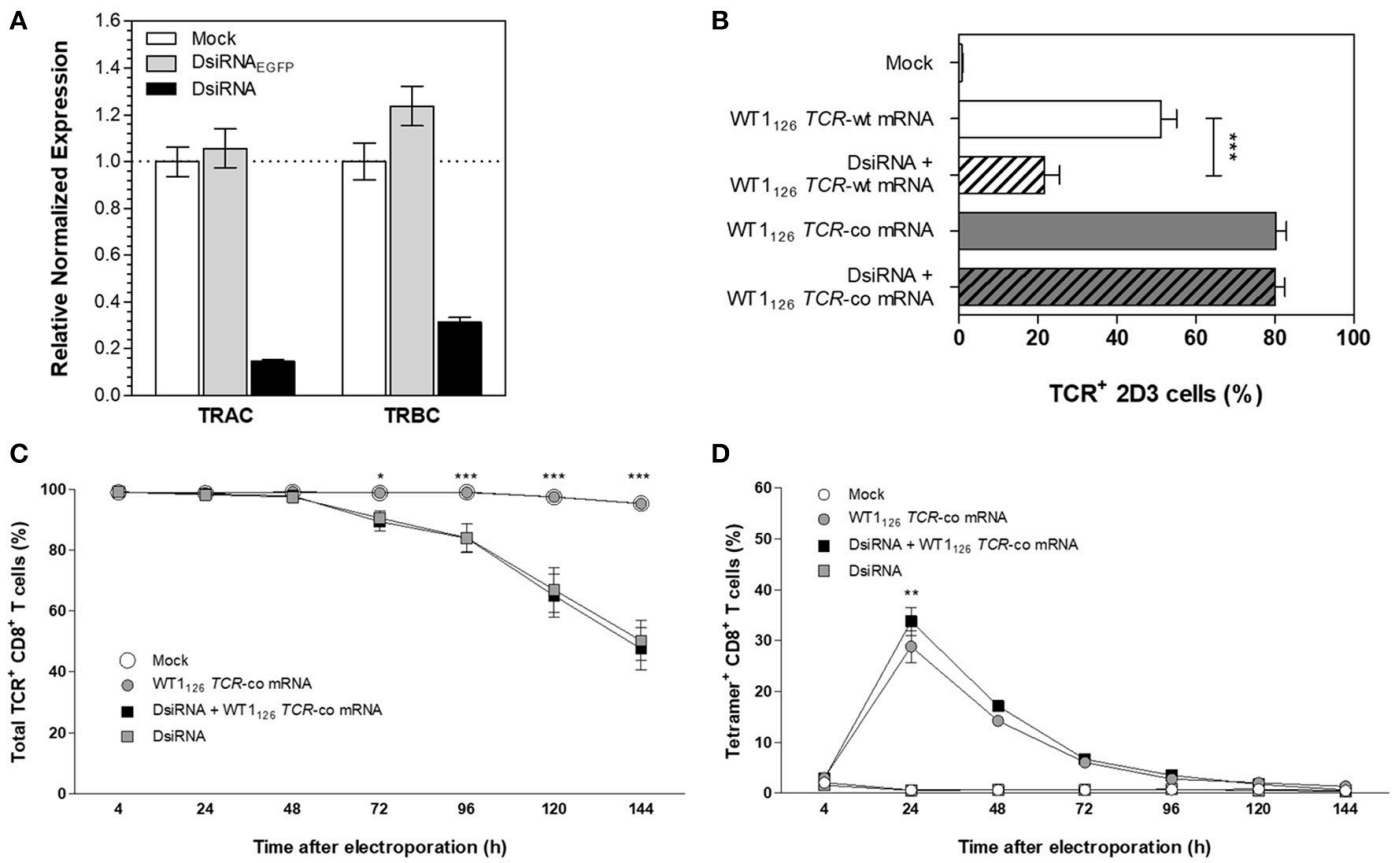
Silencing effect of DsiRNA against *TRAC* and *TRBC* upon simultaneous DsiRNA and *TCR* mRNA electroporation. **(A)** Analysis of *TRAC* and *TRBC* gene expression using RT-qPCR in Jurkat E6-1 cells after single electroporation with a control DsiRNA against *EGFP* (DsiRNA_EGFP_), with DsiRNA targeting *TRAC* and *TRBC* (DsiRNA) or no DsiRNA (mock). Expression levels were normalized to the reference genes importin-8 and ribosomal protein L13A and analyzed relative to mock electroporation. **(B)** TCR-deficient 2D3 cells were electroporated simultaneously with wild-type (-wt) or codon-optimized (-co) WT1_126_ *TCR* mRNA and DsiRNA against *TRAC* and *TRBC* or electroporated with WT1_126_ *TCR* mRNA only. TCR surface expression was analyzed 24 h after transfection (mean ± SEM of 3 replicate experiments). Primary unstimulated CD8^+^ T cells were electroporated simultaneously with WT1_126_ *TCR*-co mRNA and DsiRNA against *TRAC* and *TRBC* or with *TCR* mRNA only. The percentage of total TCR expression **(C)** and percentage of transgenic TCR expression **(D)** was measured in primary unstimulated CD8^+^ T cells at different time points after electroporation (*n* = 3; mean ± SEM). *P < 0.05; **P < 0.01; ***P < 0.001; *TRAC*, T-cell receptor alpha constant region; *TRBC*, T-cell receptor beta constant region; Mock, mock electroporation; DsiRNA_EGFP_, Dicer-substrate small interfering RNA directed against *EGFP* gene; DsiRNA, Dicer-substrate small interfering RNAs directed against *TRAC* and *TRBC* genes; WT1, Wilms' tumor 1; wt, wild-type; co, codon-optimized.

To tackle the problem of mispairing in TCR-engineered primary T cells, we focused on using RNA interference to mediate downregulation of endogenous *TCR* transcripts combined with codon-optimized *TCR* mRNA transfection. In view of the superiority of Dicer-substrate small interfering RNAs (DsiRNA) vs. canonical small interfering RNA in silencing of target mRNA (20–22), we designed DsiRNA duplexes to specifically recognize the coding sequences of wild-type *TCR* alpha (*TRAC*) and *TCR* beta (*TRBC*) constant regions (Figure 2). Thus, wild-type, but not codon-optimized *TCR* sequences would be sensitive to DsiRNA-mediated knockdown. We first analyzed suppression efficiency of *TCR*-specific DsiRNA (DsiRNA) compared to mock electroporation (Mock) and DsiRNA specific for *EGFP* (DsiRNA_EGFP_) in TCR^+^ Jurkat E6-1 cells by RT-qPCR 24 h after electroporation (Figure 2A). There was a significant, more than 6-fold decrease in *TRAC* expression and more than 3-fold decrease in *TRBC* expression when cells were electroporated with DsiRNA compared to mock electroporation (*P* ≤ 0.0001). *TRAC* and *TRBC* expression levels after DsiRNA_EGFP_ electroporation remained similar to the mock electroporation, confirming that efficient inhibition of *TCR* transcripts was only achieved by *TCR*-specific DsiRNA.

We then evaluated the specific silencing effect of DsiRNA on the transgenic wild-type *TCR* mRNA sequence and the DsiRNA-resistance of the codon-optimized *TCR* mRNA sequence in TCRαβ-deficient 2D3 cells. As shown in Figure 2B, simultaneous transfection with DsiRNA and WT1_126_ *TCR*-wt mRNA led to a substantial decrease in transgenic TCR expression 24 h after electroporation as compared to the electroporation of the WT1_126_ *TCR*-wt mRNA only (21.7 ± 3.8 and 51.2 ± 3.9%, respectively), whereas TCR levels remained stable after electroporation of WT1_126_ *TCR*-co mRNA with or without DsiRNA (80.1 ± 2.4 and 80.3 ± 2.6%, respectively). This illustrates the specificity of the DsiRNA for wild-type *TCR* sequences and shows that codon-optimization protects transgenic *TCR* mRNA sequences from degradation by our designed DsiRNA. Next, we assessed the degree of DsiRNA-mediated knockdown of endogenous TCR in purified human resting CD8^+^ T cells following simultaneous electroporation with DsiRNA and WT1_126_ *TCR*-co mRNA or electroporation with either of them alone. Significant reduction in total TCR expression was observed 3 or 4 days after electroporation in those conditions where DsiRNA was added (*P* ≤ 0.05; Figure 2C). TCR surface levels were measured up to 6 days post-transfection and declined to about 50% of total TCR levels in DsiRNA-treated cells (50.3 ± 6.6 %) compared to non-treated cells (95.4 ± 1.0%). WT1_126−134_/HLA-A^*^02:01 tetramer staining of these cells showed a significantly higher WT1_126_ TCR expression when DsiRNA was simultaneously electroporated with WT1_126_ *TCR*-co mRNA (33.8 ± 2.7%), resulting in a 17% increase in transgenic TCR expression 24 h after electroporation in comparison to electroporation of mRNA alone (28.8 ± 3.2% Figure 2D). A correlation analysis for tetramer positive and transgenic TCRα or β chain positive cells could be possible at the time that antibodies specific for the transgenic TCRα or TCRβ chains would be available.

### DsiRNA electroporation 24 H prior to *TCR* codon-optimized mRNA electroporation drastically increases transgenic TCR expression

**Figure 3:**
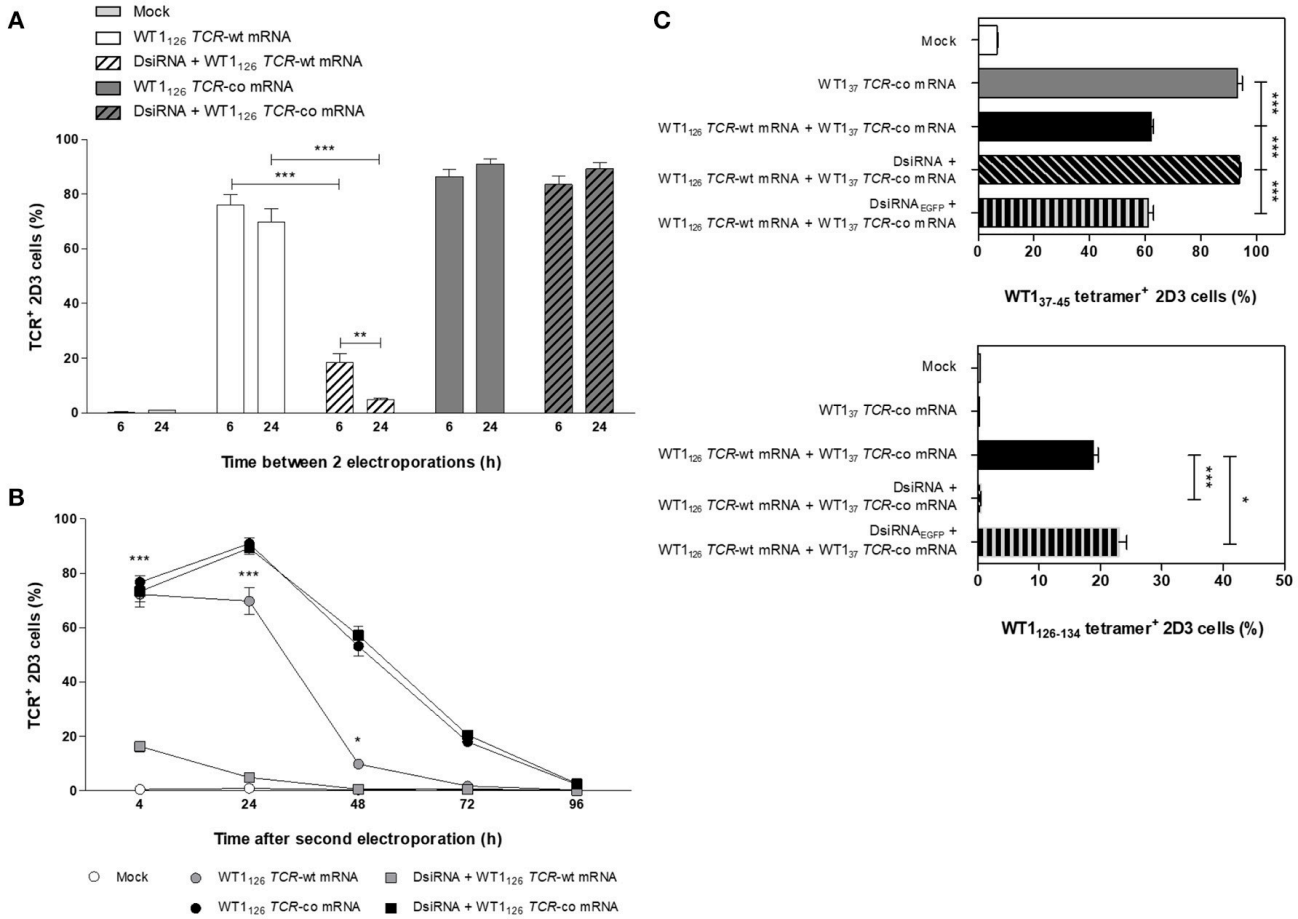
Optimization of double sequential electroporation with DsiRNA and *TCR* mRNA in 2D3 cells. **(A)** Influence of different time spans between first and second sequential electroporation on transgenic TCR expression in TCRαβ-deficient 2D3 cells. DsiRNA electroporation was performed 6 or 24 h prior to WT1_126_ *TCR* mRNA electroporation. **(B)** Kinetics of transgenic TCR expression in double sequentially-electroporated 2D3 cells. DsiRNA electroporation was performed 24 h prior to WT1_126_ *TCR* mRNA electroporation. **(C)** Effect of mispairing on transgenic TCR expression. 2D3 cells were electroporated with a DsiRNA specific for *EGFP* (DsiRNA_EGFP_) or DsiRNA for wild-type *TRAC* and *TRBC* genes (DsiRNA) 24 h before electroporation with WT1_37_ *TCR*-co mRNA or a combination of WT1_37_ *TCR*-co and WT1_126_ *TCR*-wt mRNAs. Transgenic TCR expression was analyzed 24 h after mRNA transfection with WT1_37−45_/HLA-A*02:01 tetramers (upper panel) and WT1_126−134_/HLA-A*02:01 tetramers (lower panel). All graphs show the results for 3 independent experiments (mean ± SEM). ***P < 0.001; Mock, mock electroporated; WT1, Wilms' tumor 1; wt, wild-type; co, codon-optimized; DsiRNA, Dicer-substrate small interfering RNAs directed against *TRAC* and *TRBC* genes; DsiRNA_EGFP_, Dicer-substrate small interfering RNA directed against *EGFP* gene.

To fully exploit the silencing potential of DsiRNA and to optimize transgenic TCR expression, we tested sequential electroporation of 2D3 cells with DsiRNA followed by WT1_126_ *TCR* mRNA electroporation 6 or 24 h later and analyzed TCR surface expression levels 24 h after the second electroporation (Figure 3A). Superior and significant reduction of TCR levels was observed when DsiRNA transfection was performed 24 h prior to *TCR*-wt mRNA electroporation (4.9 ± 0.5%), as compared to a 6 h interval (18.4 ± 3.3%). Kinetics of TCR expression of double sequentially-electroporated 2D3 cells with 24 h between electroporations showed a sustained and marked downregulation of TCR expression 24 h after electroporation of WT1_126_ *TCR*-wt mRNA when cells were pre-treated with DsiRNA (from 69.8 ± 4.9% to 4.9 ± 0.5% with a decrease of 93%, *P* ≤ 0.001; Figure 3B). We analyzed the degree of silencing in Jurkat E6-1 cells 24 h after DsiRNA/mock double sequential electroporation (i.e., *TRAC* and *TRBC* levels analyzed 48 h after DsiRNA electroporation) by RT-qPCR (Figure S2). We observed a significant, more than 6-fold downregulation of *TRAC* levels and more than 2-fold downregulation of *TRBC* levels compared to double sequential mock electroporation (*P* ≤ 0.01 for *TRAC* and *P* ≤ 0.05 for *TRBC*), similar to the results obtained 24 h after one electroporation with DsiRNA only (Figure 2A). These results indicate that the silencing effect of the DsiRNA on the target endogenous *TCR* transcripts is still markedly present after a second electroporation and, more importantly, that the DsiRNA still exert their effect 48 h after DsiRNA electroporation. This guarantees that TCR mispairing is being prevented when the peak of transgenic TCR expression occurs after DsiRNA/TCR-co double sequential electroporation.

To further investigate the degree of mispairing between two TCRs expressed concomitantly, we generated from another CTL clone of the same patient, a TCR reactive to the WT1_37−45_ peptide (Figure S3A) and produced the codon-optimized mRNA construct (WT1_37_ *TCR*-co mRNA; Figure S3B). Using the same optimized double sequential electroporation platform with 24 h between first and second electroporation, 2D3 cells were transfected with DsiRNA against *TRAC* and *TRBC* sequences or a control DsiRNA targeting *EGFP* mRNA prior to electroporation with WT1_37_ *TCR*-co mRNA or a combination of WT1_37_ *TCR*-co mRNA and WT1_126_ *TCR*-wt and stained with WT1_37−45_/HLA-A^*^02:01 and WT1_126−134_/HLA-A^*^02:01 tetramers 24 h after mRNA electroporation. Of note, the WT1/HLA-A^*^02:01 tetramers used to quantify WT1_37_ or WT1_126_ TCR expression cannot bind to mispaired TCRs. As shown in Figure 3C, 2D3 cells electroporated with WT1_37_ *TCR*-co mRNA expressed high levels of WT1_37_ TCR (93.0 ± 1.8%), whereas a significant reduction of 33% (*P* ≤ 0.001) was observed when WT1_126_ *TCR*-wt mRNA was co-electroporated (62.1 ± 0.9%), indicative for the degree of mispairing when two TCRs are expressed in the same cell. Importantly, complete inhibition of mispairing between the two TCRs could be achieved upon pre-transfection with DsiRNA directed against *TRAC* and *TRBC* sequences (93.7 ± 0.6%), but not DsiRNA_EGFP_ (60.9 ± 2.0%), leading to a full recovery of WT1_37_ TCR expression (*P* ≤ 0.001; Figure 3C, **upper**). Similarly, the percentage of WT1_126_ TCR positive cells was nearly abolished in cells treated with DsiRNA directed against *TRAC* and *TRBC* (0.49 ± 0.02%), but not against *EGFP* mRNA (23.0 ± 1.3%; Figure 3C, lower), demonstrating the efficacy and specificity of DsiRNA for downregulation of *TCR*-wt mRNA.

### Double sequential electroporation of DsiRNA and *TCR* codon-optimized mRNA boosts transgenic TCR expression in primary CD8^+^ T cells

**Figure 4:**
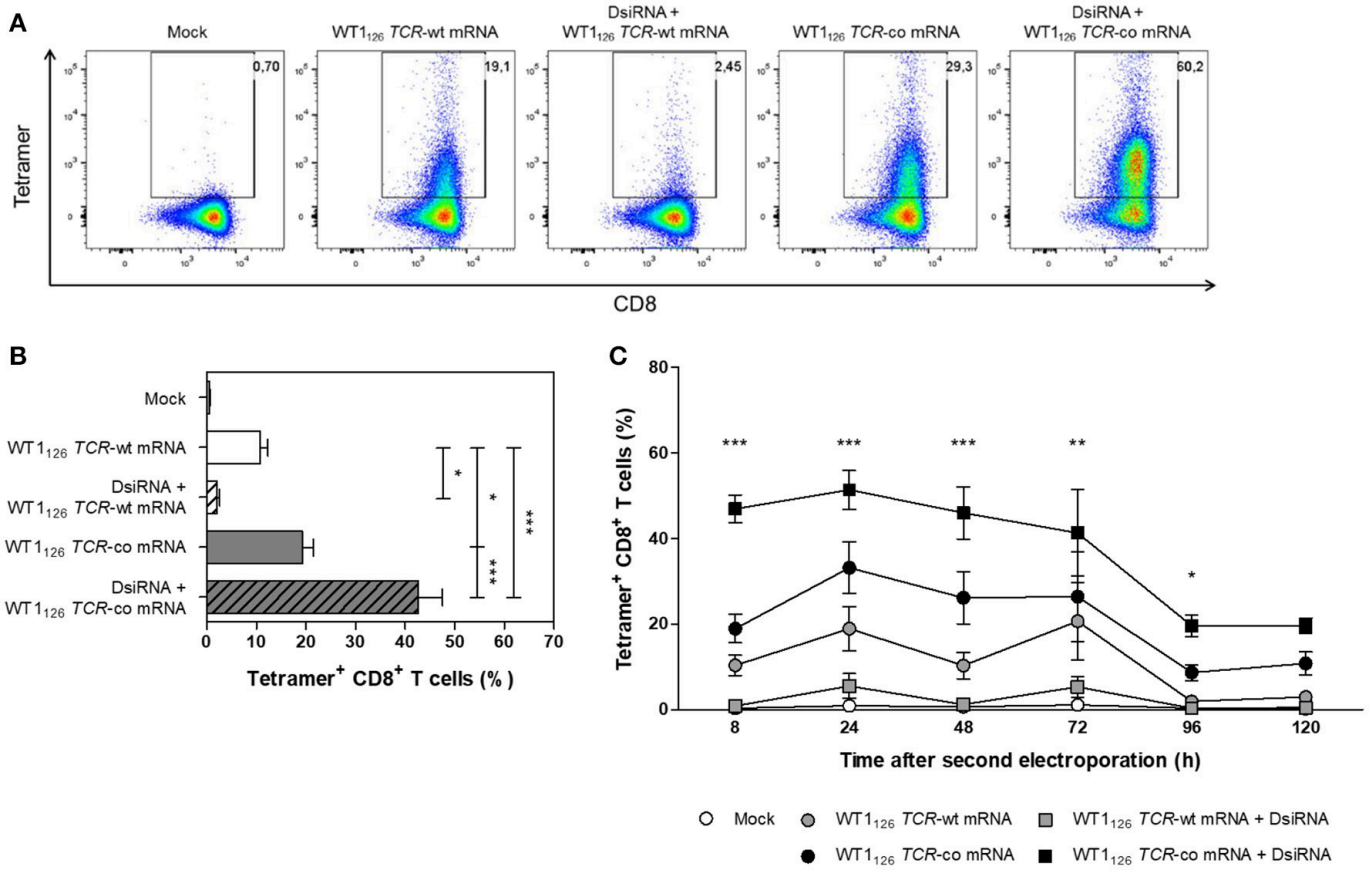
Analysis of transgene WT1_126_ TCR expression in human primary resting CD8^+^ T cells after double sequential electroporation with DsiRNA transfection performed 24 h prior to WT1_126_ *TCR* mRNA transfection. **(A)** Representative flow cytometric analysis by WT1_126−134_/HLA-A*02:01 tetramer staining 24 h after the second electroporation showing transgenic TCR expression from one out of 15 donors. The percentage of tetramer-positive CD8^+^ T cells is indicated in the upper right corner. **(B)** Transgenic TCR expression of double sequentially-electroporated resting CD8^+^ T cells was evaluated 24 h after *TCR* mRNA electroporation by WT1_126−134_/HLA-A*02:01 tetramer analysis (*n* = 15, mean ± SEM). **(C)** Kinetics of transgenic TCR expression after second electroporation of resting CD8^+^ T cells (*n* = 3, mean ± SEM). *P < 0.05; **P < 0.01; ***P < 0.001; Mock, mock electroporated; WT1, Wilms' tumor 1; wt, wild-type; co, codon-optimized; DsiRNA, Dicer-substrate small interference RNAs directed against *TRAC* and *TRBC* genes.

Next, the optimized DsiRNA + *TCR* mRNA double sequential electroporation protocol was validated in human primary resting CD8^+^ T cells from healthy donors (Figure 4). We observed a 2-fold increase in codon-optimized TCR expression using the double sequential electroporation (42.6 ± 4.9%; mean ± SEM of *n* = 15) vs. a single *TCR* mRNA electroporation (19.3 ± 2.2%; *P* ≤ 0.001; Figures 4A,B). Transgenic TCR expression was maintained for at least 5 days after WT1_126_ *TCR*-co mRNA electroporation, with superior TCR expression kinetics up until day 4 when T cells were pre-treated with DsiRNA (19.6 ± 2.5% for DsiRNA + WT1_126_ *TCR*-co mRNA vs. 8.7 ± 1.9% for WT1_126_ *TCR*-co mRNA only at day 4; Figure 4C). Gene expression analysis of endogenous *TRAC* and *TRBC* transcripts revealed that DsiRNA targeting these sequences significantly downregulated the levels of *TRAC* and *TRBC* transcripts in resting CD8^+^ T cells. Expression levels were decreased more than 3-fold compared to mock electroporation (*P* ≤ 0.01).

### Redirection of effector response of primary resting CD8^+^ T cells via DsiRNA/*TCR* mRNA double sequential electroporation promotes killing of target cells

**Figure 5:**
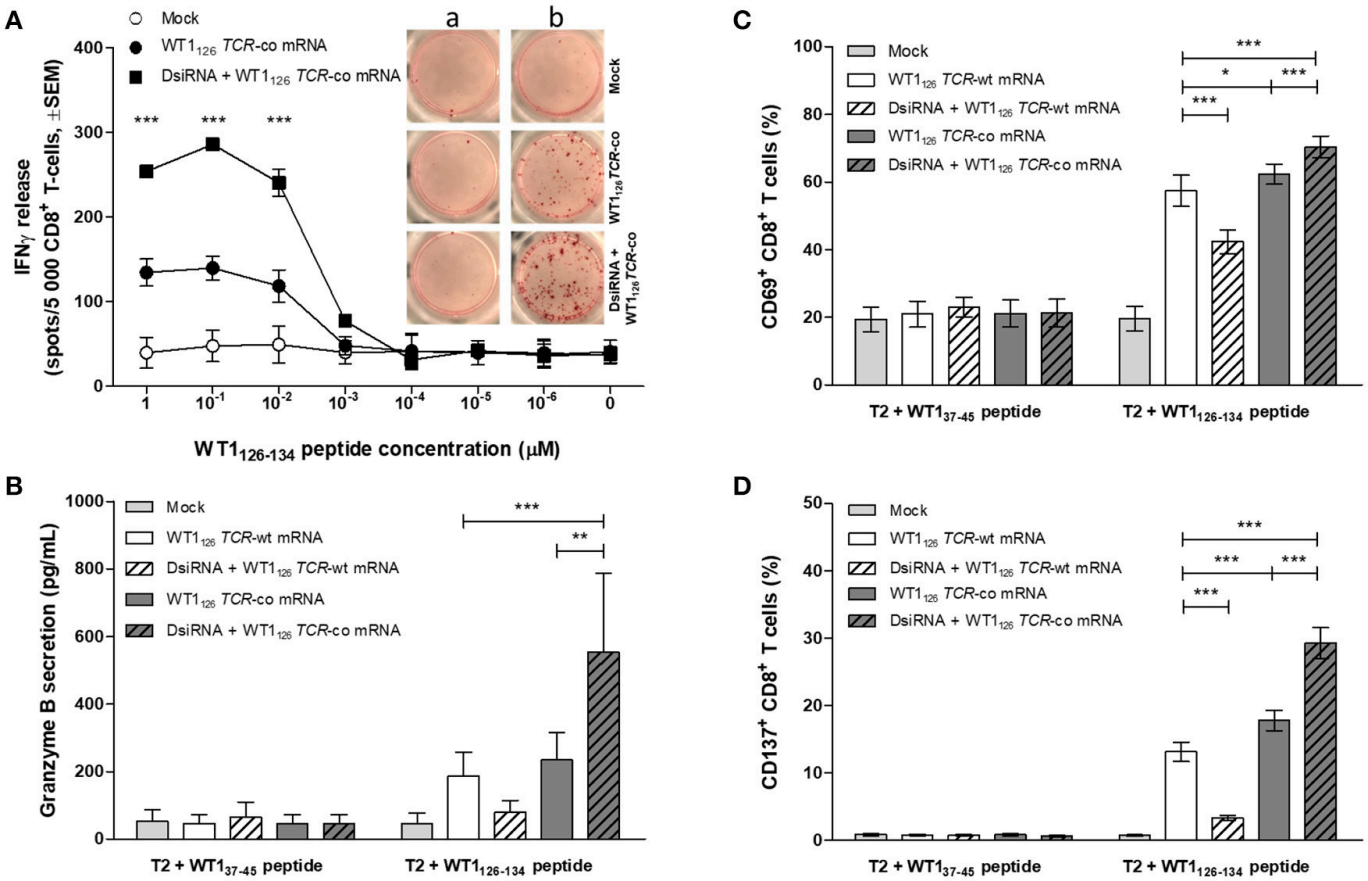
Effect of DsiRNA-mediated silencing of endogenous *TCR* on WT1_126_ TCR avidity and antigen-specific activation in resting CD8^+^ T cells after double sequential electroporation with DsiRNA transfection performed 24 h prior to WT1_126_ *TCR* mRNA transfection. **(A)** Release of IFN-γ was measured by IFN-γ ELISpot after co-culture of double sequentially-electroporated CD8^+^ T cells and T2 cells that were pulsed with decreasing concentrations of WT1_126−134_ peptide (*n* = 2, mean ± SEM). Within the graph, representative wells of co-cultures with non-peptide-pulsed T2 cells (a) or peptide-pulsed T2 cells (b, 1μM peptide). **(B–D)** Primary unstimulated CD8^+^ T cells were double sequentially-electroporated with WT1_126_ *TCR* mRNA after DsiRNA or mock (no RNA) electroporation. Transfected CD8^+^ T cells were co-cultured with peptide-pulsed T2 cells in an effector:target ratio of 4:1. After 24 h, cells were pelleted by centrifugation and supernatants were collected. **(B)** Secretion of granzyme B was analyzed in supernatants using a human granzyme B ELISA kit (*n* = 4, mean ± SEM). Flow cytometric analysis of antigen-specific T cell activation was analyzed by activation-induced upregulation of surface markers CD69 **(C)** and CD137 **(D)** in CD8^+^ T cells (*n* = 5, mean ± SEM). *P < 0.05; **P < 0.01; ***P < 0.001; IFNγ, interferon-γ; Mock, mock electroporated; WT1, Wilms' tumor 1; co, codon-optimized; DsiRNA, Dicer-substrate small interference RNAs directed against *TRAC* and *TRBC* genes.

**Figure 6:**
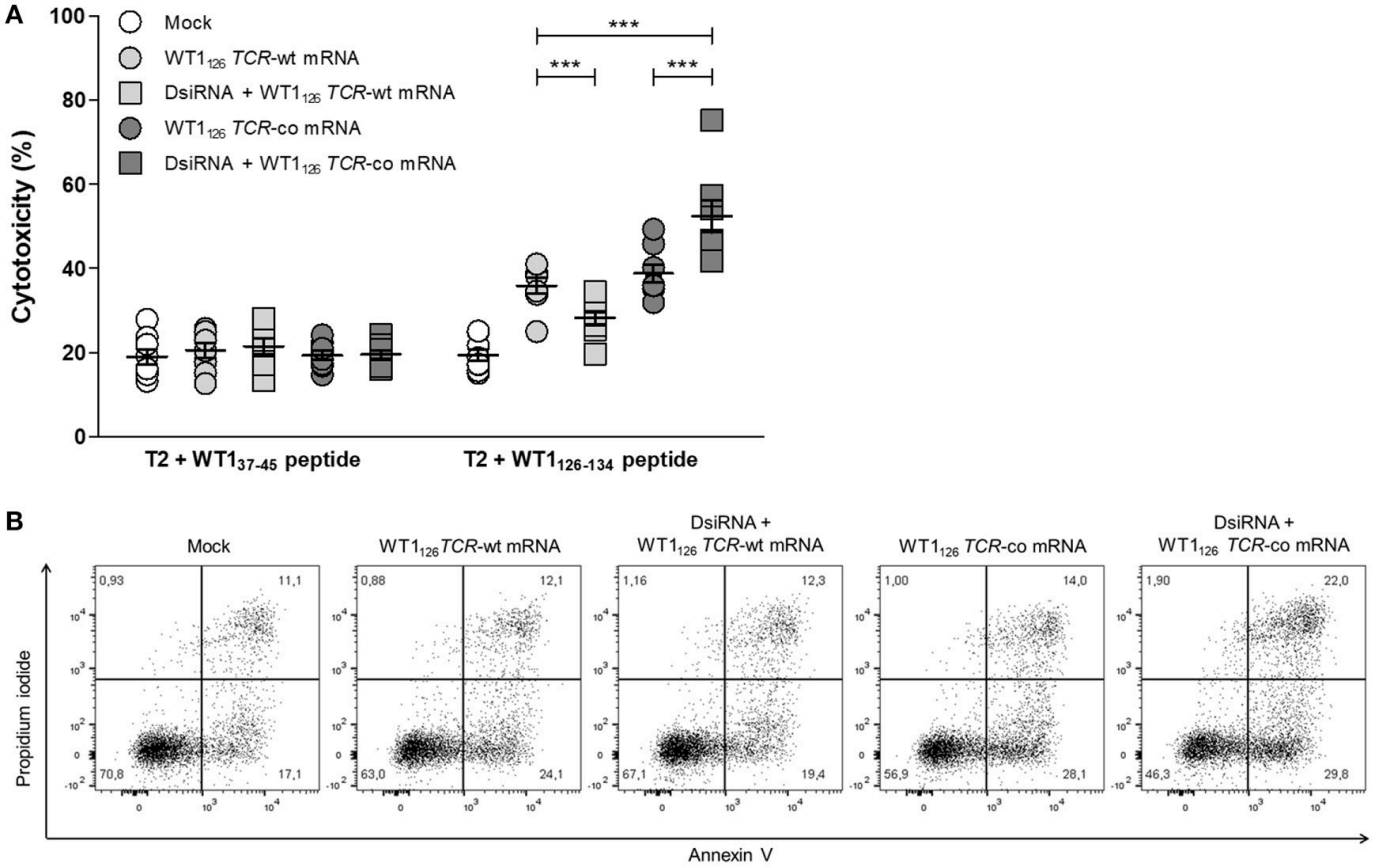
Antigen-specific cytotoxicity of primary resting CD8^+^ T cells is boosted after double sequential electroporation with DsiRNA and WT1_126_ *TCR*-co mRNA. **(A)** Cytotoxic activity of double sequentially-electroporated CD8^+^ T cells after 6 h of co-culture with peptide-pulsed T2 cells (E:T ratio = 20:1, *n* = 8, mean ± SEM). WT1_37−45_ peptide-pulsed T2 cells served as negative control target. **(B)** Representative example of WT1_126−134_ peptide-pulsed T2 cell cytotoxicity mediated by double sequentially-electroporated CD8^+^ T cells after 6 h of co-culture. The percentage of cells is indicated in each quadrant. ***P < 0.001; Mock, mock electroporated; WT1, Wilms' tumor 1; wt, wild-type; co, codon-optimized; DsiRNA, Dicer-substrate small interfering RNAs directed against *TRAC* and *TRBC* genes.

We evaluated whether the improved TCR expression after double sequential electroporation correlated with enhanced redirected T-cell effector functions (Figure 5). First, to assess the functional avidity of the TCR for its cognate peptide, electroporated CD8^+^ T cells were assayed for interferon (IFN)-γ production upon recognition of epitope-carrying target cells. DsiRNA-mediated silencing of the endogenous *TCR* mRNA in *TCR* mRNA-electroporated CD8^+^ T cells led to a significantly improved recognition of WT1_126−134_ peptide-pulsed target cells as compared to their non-silenced counterparts up to a WT1_126−134_ peptide concentration of 10^−2^ μM (*P* ≤ 0.001; Figure 5A). This activation threshold is equivalent to that observed in TCR-deficient 2D3 cells (Figure S1B). Similar results were obtained upon analysis of granzyme B secretion in supernatants of double sequentially-electroporated CD8^+^ T cells co-cultured with peptide-pulsed T2 cells (Figure 5B). In this case, pre-treatment with DsiRNA of WT1_126_ *TCR*-co mRNA electroporated T cells led to a 2.4-fold increase compared to non-treated cells (554.0 ± 232.5 pg/mL and 234.3 ± 82.0 pg/mL, respectively). Second, double sequentially-electroporated CD8^+^ T cells were analyzed for expression of activation markers CD69 and CD137 after co-culture with peptide-pulsed T2 cells (Figures 5C,D). DsiRNA-pre-treated and WT1_126_ *TCR*-co mRNA-transfected CD8^+^ T cells exhibited significantly higher frequencies of CD69 (70.4 ± 3.2%) and CD137 (29.3 ± 2.3%) positivity in an antigen-specific manner, as compared to cells that were electroporated with *TCR*-co mRNA only (62.3 ± 3.0% CD69^+^ and 17.8 ± 1.5 % CD137^+^ CD8^+^ T cells), reaching a difference of 64% for CD137. Frequencies of both CD69^+^ and CD137^+^ CD8^+^ T cells was always significantly lower when these cells were electroporated with WT1_126_ *TCR*-wt mRNA either pre-treated with DsiRNA or not (42.4 ± 3.5% vs. 57.4 ± 4.6 % for CD69 and 3.3 ± 0.4 % vs. 13.1 ± 1.4 for CD137; Figures 5C,D). Finally, we evaluated the cytotoxic capacity of transfected resting CD8^+^ T cells (Figure 6). Antigen-specific cytotoxicity by WT1_126_ *TCR*-co mRNA-electroporated CD8^+^ T cells was superior in DsiRNA pre-treated (52.4 ± 3.8%) as compared to non-pre-treated CD8^+^ T cells (38.8 ± 2.1%), whereas it was virtually reduced to mock levels in DsiRNA-pre-treated WT1_126_ *TCR*-wt mRNA-electroporated T cells (28.3 ± 1.6%; Figures 6A,B). There was no significant difference in mean levels of cytotoxicity after wild-type or codon-optimized mRNA transfection without DsiRNA pre-treatment (35.9 ± 1.9 and 38.8 ± 2.1%, respectively).

## Discussion

In recent years, different strategies to improve TCR gene transfer and functionality in T cells have been developed. Advances in this field include retro- and lentiviral transduction protocols to achieve stable and long-term TCR expression, modification of TCR affinity, incorporation of cysteine bonds or murinization of constant regions to enhance TCR pairing and conjugation of TCRs with co-stimulatory signals (23). However, clinical safety issues, complex TCR manipulations and high costs associated with these methods are an obstacle for widespread clinical use. Here, we describe a double sequential electroporation procedure with DsiRNA and codon-optimized *TCR* mRNA for rapid TCR engineering of T cells. This non-viral and non-genotoxic approach results in robust transient expression of transgenic TCR and superior T-cell effector function of primary resting CD8^+^ T cells while preventing TCR mispairing by DsiRNA-mediated silencing of endogenous TCR.

Regardless of the origin of wild-type *TCR* sequences (endogenous or WT1_126_ *TCR*-wt mRNA) and of T cells (Jurkat cell lines or primary CD8^+^ T cells), electroporation of DsiRNA reproducibly led to a reduction in TCR expression from these wild-type sequences. In contrast, DsiRNA transfection enhanced the surface expression of transgenic TCR after electroporation with codon-optimized *TCR* mRNA in the presence of a wild-type and/or endogenous *TCR* mRNA. This confirms the specificity of the DsiRNA for wild-type TCR sequences and the reduction of TCR mispairing. Using this strategy, production of TCR-engineered T cells is greatly simplified and broadly applicable because codon optimization is a commonly available tool and because the particular design of the DsiRNA will allow the suppression of any endogenous TCR. This method should avoid the need for more complex TCR modifications in order to improve transgenic TCR pairing.

In general, mRNA electroporation is one of the methods of choice for non-viral transfection of immune cells, including dendritic cells (14) and T cells (15), and can be adopted for TCR engineering of primary unstimulated T cells as demonstrated in this and other studies (24, 25). Thus, resting T cells can be transfected and antigen-activated without the need for pre-activation culture protocols. This is an advantage for clinical T cell therapy purposes, as it considerably cuts production time and costs.

Since simultaneous electroporation of DsiRNA and codon-optimized *TCR* mRNA produced a low percentage increase in TCR levels we aimed to improve expression of transgenic TCR by transfecting DsiRNA prior to codon-optimized *TCR* mRNA electroporation. During the optimization of the double sequential electroporation, best results were obtained with a 24 h interval between DsiRNA and codon-optimized *TCR* mRNA electroporation, pointing to possible overlapping kinetics of transgenic TCR expression and DsiRNA-mediated silencing (26) of endogenous TCR. Therefore, 24 h double sequential electroporation provides a time window for DsiRNA assembly with RNA-induced silencing complex (RISC), RISC activation and downregulation of endogenous *TCR* mRNA before introducing a new *TCR* mRNA. Importantly, the DsiRNA-mediated silencing of the endogenous TCR is markedly present at least until the point when transgenic TCR expression from codon-optimized TCR mRNA is at its highest levels, ensuring that TCR mispairing is avoided when the T cells display their maximum functionality.

With regards to other silencing strategies, levels of transgenic TCR expression after treatment with DsiRNA were comparable, if not higher, to those obtained by retroviral transduction of constructs containing short hairpin RNA (shRNA) or clusters of primary microRNAs (pri-miRNAs) and siRNA-resistant antigen-specific TCR (27, 28). In a preclinical study using these shRNA-containing retroviral vectors, Ochi and collaborators (29) reported that transduced CTLs from leukemia patients showed high antileukemic responses against autologous tumor cells *in vitro* as well as *in vivo* in a mouse xenograft model, providing evidence that silencing of endogenous TCR is a powerful tool for T cell-based cancer immunotherapy. Other non-viral approaches have also been exploited for the transfection of T cells, such as the electroporation of a DNA plasmid integrating the *Sleeping Beauty* transposon/transposase system (30, 31). However, as occurs with integrating viral vectors, the risk of insertional mutagenesis is an important element to consider for its clinical application. Compared to these strategies to express different forms of RNA interference or to introduce a transgenic TCR, we show here that double sequential electroporation with DsiRNA and *TCR* mRNA is an efficient non-integrating system that rapidly redirects and boosts T-cell effector function. This highlights the potential efficacy of this immunotherapy for clinical trials. Electroporation provides a time window in which transgenic TCR is present and engineered CTLs will recognize the antigen of interest, followed by natural degradation of transfected DsiRNA and mRNA and restitution to their previous TCR phenotype.

With our method, the introduction of DsiRNA increases transgenic TCR expression, yet the duration of transgenic *TCR* expression remains the same with or without DsiRNA. We showed the kinetics of the surface expression of the antigen-specific TCRs on viable cells after double sequential electroporation. Since we worked with unactivated T cells directly from PBMC after CD8^+^ T cell isolation, the viability and life span of these cells will be limited unless growth factors to promote T cell survival are added to the culture medium. In our experiments, there was no pre-activation of T cells nor addition of cytokines. Therefore, TCR expression on viable cells was measured up to 5–6 days after transfection, time in which T cell viability was naturally declining due to the absence of survival signals. By doing so, we prevented the introduction of any confounding factors that might had influenced the kinetics of the TCR expression. Consequently, because of the transient nature of the electroporated DsiRNA and mRNA, one single administration may not be enough for clinical effect in large-scale clinical trials. To ensure the effectiveness of our approach repeated administrations of TCR-engineered T cells would be required in order to avoid T-cell expansion protocols. Then, the isolated T cells can be cryopreserved in different aliquots (TCR-engineered or not) for later use, outrivaling other methods by its rapid production for administration to patients. Time-limited expression of transgenic TCR also favors the possibility of testing the safety of a particular transgenic TCR and the potential presence of “off-target” specificities from the introduced TCR in phase I studies prior to trials with more stable and expensive expression systems and/or complete disruption of endogenous TCR sequences (32). In the case of the specificity of the DsiRNA, it is worth mentioning that the DsiRNA used in this study were analyzed for their specificity against the wild-type or codon-optimized *TRAC* and *TRBC* transcripts. In the event of any potential off-target effects eliciting the downregulation of other non-targeted mRNAs, the transient nature of the electroporated DsiRNA will prevent the development of long-lasting adverse effects.

As for any other immunotherapy, CTL dosage and frequency of injections will have to be tested to ascertain the efficacy of one or more administrations. To this end, the field of chimeric antigen receptors (CAR)-modified T cells has also been exploiting mRNA electroporation in the last years (33–38) with different results. With regards to dosage and efficacy, Barrett et al. (34) showed that human mRNA-electroporated CD19-specific CAR-engineered CTLs had potent *in vitro* antileukemic killing activity against CD19^+^ cell lines and reduced disease burden within 1 day after injection of a single dose in a mouse model xenografted with human CD19^+^ leukemia cells. In another study from the same group (38), mice injected with primary leukemia cells were given multiple doses of anti-CD19 CAR T cells engineered either via electroporation or lentiviral transduction. Repeated injections of mRNA-electroporated CAR-engineered CTLs combined with lymphodepletion achieved similar results when compared to stable lentiviral transduction, emphasizing the applicability and efficiency of RNA-engineered T cells for the clinic. In fact, clinical trials have been conducted to study the efficacy and safety of mRNA-transfected CAR T cells for the treatment of cancer (39, 40, 41), underscoring the importance of transient systems to test possible toxicities prior to more stable approaches. On this subject, although infusion of mRNA-electroporated CAR T cells was well tolerated in general and serious adverse effects were not or possibly not related to the study drug, one patient showed anaphylaxis with production of IgE antibodies against murine antibody-derived antigen binding domain of the mesothelin-specific CAR (39).

Finally, combining TCR engineering with blockade of immune checkpoint proteins, such as programmed cell death protein-1 (PD-1) (42), is an appealing strategy to redirect CTL specificity while reducing PD-1-induced anergy (43, 44). For instance, Iwamura et al. (45) showed that antigen-stimulated T cells expressed programmed death-ligand (PD-L) 1 and 2 and that electroporation of siRNA to downregulate expression of PD-L1/2 combined with retroviral transduction of a melanoma-specific TCR resulted in increased effector function against MAGE-A4^+^ cells. Thus, the combination of DsiRNAs specific for immune checkpoint inhibitors and for the endogenous *TCR* chains in a double sequential electroporation system could be further analyzed to maximize the success of cancer treatments.

In conclusion, we generated a novel non-viral and non-genotoxic platform for efficient T-cell receptor engineering for the development of a safer, faster and cost-effective adoptive T-cell therapy. Electroporation of T lymphocytes with DsiRNA prior to electroporation of codon-optimized *TCR* mRNA leads to robust expression of introduced TCR while inhibiting TCR mispairing and results in superior functionality of TCR-engineered cells. In our view, these results warrant further *in vivo* validation of this promising non-integrating, efficient and affordable system to safely TCR engineer T cells for clinical trials.

## Materials and methods

### Study design

The hypothesis of this study was that sequential electroporation of wild type TCR-specific DsiRNA and codon-optimized *TCR* mRNA would improve transgenic TCR expression by silencing of endogenous *TCR* transcripts *in vitro*. We tested transfection of DsiRNA and *TCR* mRNA using cell lines and primary samples from anonymous healthy donors provided by the Blood Service of the Flemish Red Cross (Mechelen, Belgium), following the approval of the Ethics Committee of the Antwerp University Hospital and the University of Antwerp (Antwerp, Belgium) under reference number 16/35/357. Information regarding number of replicates can be found in the figure legends. Validation of the specificity and efficacy of DsiRNA and optimization of double sequential electroporation were performed using cell lines that endogenously express *TCR* or by electroporation of wild-type *TCR* mRNA. Epitope-specific T cell effector function was analyzed by co-culture of cells with a tumor cell line in the presence of relevant or irrelevant peptides.

### T cell isolation and cell lines

Peripheral blood mononuclear cells (PBMCs) from anonymous healthy donors were separated from whole blood using Ficoll density gradient centrifugation (Ficoll-Paque PLUS; GE Healthcare). Cytotoxic CD8^+^ T cells were positively selected using human CD8 magnetic microbeads (Miltenyi Biotec), following manufacturer's instructions. Isolated CD8^+^ T cells were then used in electroporation experiments and were considered to be in a resting phase since no pre-activating treatment was applied. Purity of isolated CD8^+^ T cells was analyzed by staining with anti-human CD3-PerCP, CD4-PE and CD8-FITC or matched isotype control monoclonal antibodies (mAbs; BD Biosciences). Samples were measured on a FACScan flow cytometer (BD Biosciences). The human acute T cell leukemia cell lines Jurkat Clone E6-1 (ATCC, TIB-152) and 2D3 (46) were maintained in Roswell Park Memorial Institute 1640 (RPMI) culture medium (Gibco Invitrogen) supplemented with 10% fetal bovine serum (FBS; Gibco Invitrogen). 2D3 cells were generated from TCRαβ-deficient Jurkat 76 cells by transduction with human *CD8 alpha-E2A-CD8 beta* construct (both Jurkat 76 cells and CD8-encoding plasmid were kind gifts of Prof. Hans Stauss, Institute of Immunity and Transplantation, University College London, London, UK) and with a plasmid vector containing the enhanced green fluorescent protein (*EGFP*) gene under the control of a nuclear factor of activated T-cell (NFAT) promoter (NFAT-EGFP plasmid kindly provided by Prof. Takashi Saito, Riken Research Center for Allergy and Immunology, Yokohama, Japan). HLA-A^*^02:01-positive T2 cells, a human lymphoblastoid cell line with transporter associated with antigen presentation (TAP) deficiency that can be loaded with exogenous MHC class I-restricted peptides, were kindly provided by Dr. Pierre Van der Bruggen (Ludwig Institute for Cancer Research, Brussels, Belgium) and were maintained in Iscove's Modified Dulbecco's Medium (IMDM; Gibco Invitrogen) supplemented with 10% FBS. Cell lines were maintained in logarithmic growth phase at 37°C in a humidified atmosphere supplemented with 5% CO_2_.

### Cloning of WT1_37−45_- and WT1_126−134_-specific TCR genes and vector construction

WT1_37−45_ and WT1_126−134_-specific CTL clones were established from an AML patient (UPN08) (12) by single-cell sorting of WT1_37−45_/HLA-A^*^02:01 or WT1_126−134_/HLA-A^*^02:01 tetramer-positive CTLs. Briefly, frozen PBMCs were thawed and stained with 7-AAD (eBioscience), WT1/HLA-A^*^02:01 PE-labeled tetramers (Medical & Biological Laboratories Co.), anti-human CD3-Pacific Blue (clone UCHT1) and CD8-APC-Cy7 (clone SK1) mAbs (BD Biosciences) and single-cell sorting was performed using FACSAria (BD Biosciences). The sorted cells were expanded by co-culture with irradiated allogeneic PBMCs in the presence of interleukin (IL)-2 (100 IU/ml; Shionogi & Co., Ltd.) and phytohemagglutinin (PHA; Remel Inc., 2 μg/ml) in a 96-well round-bottom plate. Expanded CTL clones were screened for WT1_37−45_ or WT1_126−134_ specificity by tetramer staining or intracellular cytokine assay. WT1-specific *TCR*α and *TCR*β genes from established clones were isolated by a 5′-RACE PCR method and identified by the International Immunogenetics Information System (http://www.imgt.org/IMGT_vquest/vquest?livret=0&Option=humanTcR) as described previously (47). The cloned wild type (wt) *TCR*α and *TCR*β genes were linked with the 2A sequence from porcine teschovirus-1 (P2A) (18) and cloned into the Spe I-Xho I site of pST1 plasmid (48, 49) (WT1_126_ *TCR*-wt, Figure 1B). The pST1 WT1_126_ *TCR*-co vector was derived from the pST1 WT1_126_ *TCR*-wt vector by codon-optimization of the WT_126_ *TCR*-wt sequence and insertion of *TCR*β before the 2A peptide sequence (28) (WT1_126_ *TCR*-co, Figure 1B). For the WT1_37−45_-specific TCR, only the pST1 WT1_37_ *TCR*-co vector containing the codon-optimized TCR was generated (WT1_37_ *TCR*-co, Figure S3).

### *in vitro* mRNA transcription

SoloPack Golden supercompetent *E. coli* cells were transformed with pST1 DNA plasmids according to manufacturer's instructions. Transformed *E. coli* cells were cultured in LB-kanamycin agar plates and incubated overnight at 37°C and amplified in LB-kanamycin cultures at 37°C under constant motion. Plasmid DNA isolation and purification from bacterial cells were performed using the Nucleobond Xtra Midi EF and Nucleobond finalizer kits (Macherey-Nagel). Next, plasmid DNAs were digested with Sap-I restriction enzyme (Thermo Fisher Scientific) for 16 h at 37°C. Capped mRNA transcripts were synthesized from linearized plasmids and purified by DNase digestion and LiCl precipitation using a mMessage mMachine T7 *in vitro* transcription kit (Life Technologies) following manufacturer's recommendations.

### Single electroporation

Before electroporation, 10 × 10^6^ viable 2D3 or human primary unstimulated CD8^+^ T cells were washed twice in cold serum-free Opti-MEM I medium (Gibco Invitrogen), resuspended in 200 μL of the same medium and transferred to a 4.0 mm electroporation cuvette (Cell Projects). Next, one microgram of *in vitro* transcribed mRNA per 10^6^ cells and/or a 100 μM pool of two DsiRNA against the wild-type sequences of the T-cell receptor constant alpha and beta regions (*TRAC* and *TRBC*) in a 1:1 ratio, or a control DsiRNA against *EGFP* (Integrated DNA Technologies) were added to the cuvette. Electroporations were performed in a Gene Pulser Xcell™ device (Bio-Rad Laboratories) using Square Wave protocol (500 V, 5 ms, 0 gap, 1 pulse). As a negative control, cells were electroporated under the same conditions without the addition of any RNA (“Mock”). Immediately after electroporation, cells were transferred to 5 mL of RPMI medium supplemented with 10% FBS (2D3 cells) or AIM-V medium (Gibco Invitrogen) with 10% human AB serum (Gibco Invitrogen) (CD8^+^ T cells) and incubated for a minimum of 20 min at 37°C and 5% CO_2_ prior to analysis. For further analysis, cells were centrifuged and resuspended in RPMI supplemented with 5% FBS (Jurkat E6-1 or 2D3 cells) or AIM-V medium with 5% human AB serum (primary CD8^+^ T cells).

### Double sequential electroporation

Similar to single electroporation of DsiRNA, 10 × 10^6^ viable Jurkat E6-1, 2D3 or human primary unstimulated CD8^+^ T cells were electroporated with 100 μM pool of two DsiRNA against the wild-type sequences of the T-cell receptor constant alpha and beta regions (*TRAC* and *TRBC*) in a 1:1 ratio, with a control DsiRNA against *EGFP* or mock electroporated (no addition of RNA) using the same settings applied for single electroporations. Immediately after electroporation, cells were transferred to 5 mL of RPMI medium supplemented with 10% FBS (Jurkat E6-1 or 2D3 cells) or AIM-V medium (Gibco Invitrogen) with 10% human AB serum (Gibco Invitrogen) (CD8^+^ T cells) and incubated for a minimum of 20 min at 37°C and 5% CO_2_. After incubation, cells were transferred to 6-well plates (Greiner Bio-one) and incubated at 37°C and 5% CO_2_. Twenty-four hours after first electroporation, cells were harvested and analyzed for cell concentration and viability. Then, samples were washed twice with cold serum-free Opti-MEM I medium (Gibco Invitrogen), resuspended in 200 μL of the same medium and transferred to a 4.0 mm electroporation cuvette (Cell Projects). Next, none (mock) or 1 μg of *in vitro* transcribed mRNA per 10^6^ viable cells was added to the cuvette. Cells were electroporated using the abovementioned settings. For the optimization of the double sequential electroporation, 2D3 cells were also incubated for 6 h after first electroporation and prior to the second electroporation. Yield 24 h after the second electroporation ranged from approximately 60–70% cells from the total primary CD8^+^ T cells before electroporation, with an average viability of 87% after the second electroporation. For further analysis, cells were centrifuged and resuspended in RPMI supplemented with 5% FBS (Jurkat E6-1 or 2D3 cells) or AIM-V medium with 5% human AB serum (primary CD8^+^ T cells).

### Analysis of transgenic tcr surface expression

2D3 cells were harvested after electroporation and stained with the following mAbs: anti-human anti-pan TCRαβ-PE (clone BW242/412; Miltenyi Biotec), CD3-PerCP (clone SK7), CD8-FITC (clone SK1) or isotype control mAbs (BD Biosciences) for 30 min at 4°C. After washing, samples were resuspended in 200 μL of FACS buffer (FACSFlow sheath fluid, BD Biosciences; 0.1% bovine serum albumin (BSA), Sigma-Aldrich; 0.05% sodium azide, Merck) and measured on a FACScan flow cytometer (BD Biosciences). Alternatively, 2D3 cells were incubated with WT1_37−45_/HLA-A^*^02:01 tetramer-APC and WT1_126−134_/HLA-A^*^02:01 tetramer-PE (monomers kindly provided by Prof. D. A. Price, Division of Infection and Immunity, Cardiff University School of Medicine, Cardiff, UK) for 30 min at 37°C, washed and stained with anti-human CD8-Pacific Blue (clone 3B5; Life Technologies), CD3-PerCP-Cy5.5 (clone UCHT1) mAbs (BD Biosciences), and LIVE/DEAD fixable aqua dead cell stain kit (Thermo Fisher Scientific) for 30 min at 4°C. After washing, cells were resuspended in 200 μL of FACS buffer for flow cytometric analysis using a FACSAria II flow cytometer (BD Biosciences). Human primary resting CD8^+^ T cells were harvested after electroporation at different time points and stained with WT1_126−134_/HLA-A^*^02:01 tetramer-PE for 30 min at 37°C. Next, cells were washed in FACS buffer and stained with anti-human CD3-PerCP (clone SK7), CD8-FITC (clone SK1) mAbs (BD Biosciences), and LIVE/DEAD fixable aqua dead cell stain kit (Thermo Fisher Scientific) for 30 min at 4°C. After washing, cells were resuspended in 200 μL of FACS buffer for flow cytometric analysis using a FACSAria II flow cytometer (BD Biosciences).

### RT-qPCR analysis

Twenty-four hours after one or two electroporations, total RNA was extracted from Jurkat E6-1 cells or human primary resting CD8^+^ T cells using RNeasy Micro kit (QIAGEN), according to the manufacturer's instructions. Complementary DNA (cDNA) was synthetized by reverse transcription from total RNA samples using iScript cDNA synthesis kits (Bio-Rad) and diluted in water to a final concentration of 5 ng/μL. Real-time PCR reactions were performed in duplicate or quadriplicate on a CFX96™ real-time PCR detection system (Bio-Rad) using SsoAdvanced ™ Universal SYBR® Green Supermix (Bio-Rad) and PrimePCR™ primers (Bio-Rad) to detect and quantify the relative abundance of T-cell receptor alpha constant region (*TRAC*; forward primer: 5′-CTGTCTGCCTATTCACCGATT-3′, reverse primer: 5′-GTCAGATTTGTTGCTCCAGG-3′) and T-cell receptor beta constant region (*TRBC*; forward primer: 5′-GGTGAATGGGAAGGAGGTG-3′, reverse primer: 5′-GTATCTGGAGTCATTGAGGGC-3′) transcripts. Importin-8 (IPO8, Hs.505136) and ribosomal protein L13A (RPL13A, Hs.523185) were chosen as reference genes (50). Results were analyzed using CFX Manager (v3.1, Bio-Rad).

### Avidity testing of the peptide-specific TCR

2D3 cells were used to analyze the avidity for the cognate peptide and functionality of the TCR after cloning. Briefly, T2 cells were pulsed with WT1_126−134_ peptide (JPT Peptide Technologies) at decreasing concentrations of a 10-fold serial dilution for 90 min at room temperature under constant motion. Electroporated 2D3 cells were cultured with peptide-pulsed T2 cells at an effector-target ratio of 2:1 for 5 h. After incubation, cultures were stained with anti-human CD3-PerCP (clone SK7) and CD8-PE mAbs (clone SK1; BD Biosciences) for 30 min at 4°C, washed and resuspended in FACS buffer. Recognition of peptide-pulsed T2 cells was analyzed by TCR activation-mediated EGFP expression using a FACScan flow cytometer (BD Biosciences).

### IFN-γ ELIspot

Antigen recognition of TCR-specific peptide-pulsed T2 cells by electroporated human primary resting CD8^+^ T cells was analyzed using human IFN-γ ELISpot basic kit (Mabtech) following manufacturer's recommendations. T2 cells were pulsed with different concentrations of a 10-fold serial dilution of WT1_126−134_ peptide for 90 min at room temperature under constant motion. For the co-cultures, 5 × 10^3^ electroporated CD8^+^ T cells per well were added to 3 × 10^4^ peptide-pulsed T2 cells per well in 0.45 μm hydrophobic Immobilon-P PVDF membrane 96-well plates (Merck Millipore). Plates were incubated overnight at 37°C and 5% CO_2_, developed and assessed on an AID ELISpot reader system (AID Autoimmun Diagnostika). Spot-forming cells (SFC) were analyzed using AID ELISpot Software version 4.0.

### Cytotoxicity assay

The killing capacity of electroporated human primary resting CD8^+^ T cells against T2 cells was determined using a flow cytometry-based protocol as described previously with minor modifications (51). Briefly, prior to co-culture tumor cells were stained with PKH67 green fluorescent cell linker dye (Sigma-Aldrich) according to the manufacturer's protocol. PKH67^+^ T2 cells were incubated with WT1_37−45_ or WT1_126−134_ peptide (JPT Peptide Technologies) in AIM-V medium (Gibco Invitrogen) for 90 min at room temperature under constant motion. Next, T2 cells were cultured alone or with electroporated human primary resting CD8^+^ T cells for 6 h at an effector-target ratio of 20:1. After co-culture, samples were stained with propidium iodide (PI) and APC-labeled annexin V (BD Biosciences). Samples were analyzed using a FACSAria II flow cytometer (BD Biosciences). Cytotoxicity was calculated based on the survival of PKH67^+^ T2 cells using the following equation:

$$\begin{array}{l} \%\text{ ​​​​​​}Cytotoxicity\\ \;=100\;-\left[\left(\frac{\frac{\%\;annexin\;V^{-}\;PI^{-}\;T2\;cells}{co-cultured\;with\;CD8^{+}\;T\;cells}}{\frac{\%\;annexin\;V^{-}\;PI^{-}\;T2\;cells}{cultured\;without\;CD8^{+}T\;cells}}\right)\times 100\right]\; \end{array}$$

### Flow cytometric analysis of activation markers

For the analysis of TCR specificity, 1 × 10^6^ T2 cells were peptide-pulsed with 10 μg/mL of WT1_37−45_ or WT1_126−134_ peptide (JPT Peptide Technologies) in 1 mL of AIM-V medium (Gibco Invitrogen) for 90 min at room temperature under constant motion. Next, T2 cells were washed and resuspended in AIM-V medium with 5% human AB serum and added to electroporated human primary resting CD8^+^ T cells at an effector-target ratio of 4:1 and incubated for 20 h at 37°C and 5% CO_2_. After incubation, supernatants were collected for analysis of cytokine secretion and cells were stained with anti-human CD8-Pacific Blue (clone 3B5; Life Technologies), CD3-PerCP-Cy5.5 (clone UCHT1), CD14-FITC (clone MφP9), CD19-FITC (clone 4G7), CD69-APC-Cy7 (clone FN50), CD137-PE (clone 4B4-1) mAbs (BD Biosciences), and LIVE/DEAD fixable aqua dead cell stain kit (Thermo Fisher Scientific) for 30 min at 4°C. Cells were washed and analyzed using a FACSAria II flow cytometer (BD Biosciences).

### Cytokine secretion assays

Secretion of IFN-γ and granzyme B by electroporated human primary resting CD8^+^ T cells was determined by enzyme-linked immunosorbent assay kits (ELISA; respectively, Peprotech, Affimetrix and R&D Systems) following manufacturer's instructions in supernatants of co-cultures used for the analysis of activation markers. All ELISA plates were measured using a Victor 3 multilabel plate reader (Perkin Elmer).

### Statistical analysis

Flow cytometry data were analyzed using FlowJo software (v10.2, TreeStar Inc). Prism software (v5, GraphPad) was used for graphing and statistical calculations. Data were analyzed using repeated measures one-way or two-way analysis of variance (ANOVA) followed by Bonferroni *post-hoc* comparisons between different electroporation conditions. Results were considered statistically significant when *P*-value was less than 0.05.

## Author contributions

FF established the 2D3 cell line and the CTL clones and cloned constructs containing the *TCR* genes. JVdB and DCD validated *TCR* mRNA using 2D3 cells. DCD conceived the method of DsiRNA pre-electroporation. DCD, JVdB, ZB, and VVT designed experiments. DCD performed experiments and analyzed data. HDR provided technical assistance. DCD, FF, EL, ZB, and VVT wrote the manuscript. EL, ES, HG, HS, ZB, and VVT supervised the project. All authors commented on and revised the manuscript.

### Conflict of interest statement

The Department of Cancer Immunology at Osaka University Graduate School of Medicine is a department in collaboration with Otsuka Pharmaceutical Co., Ltd., and is supported with a grant from the company. The remaining authors declare that the research was conducted in the absence of any commercial or financial relationships that could be construed as a potential conflict of interest.
