# Supplementary material for: Efficient and Non-genotoxic RNA-Based Engineering of Human T Cells Using Tumor-Specific T Cell Receptors With Minimal TCR Mispairing
Source: Front Immunol. 2018 Nov 7;9:2503. doi: 10.3389/fimmu.2018.02503 (PMC6234959; doi:10.3389/fimmu.2018.02503)
Supplement: Supplementary file 1 [file Data_Sheet_1.docx]

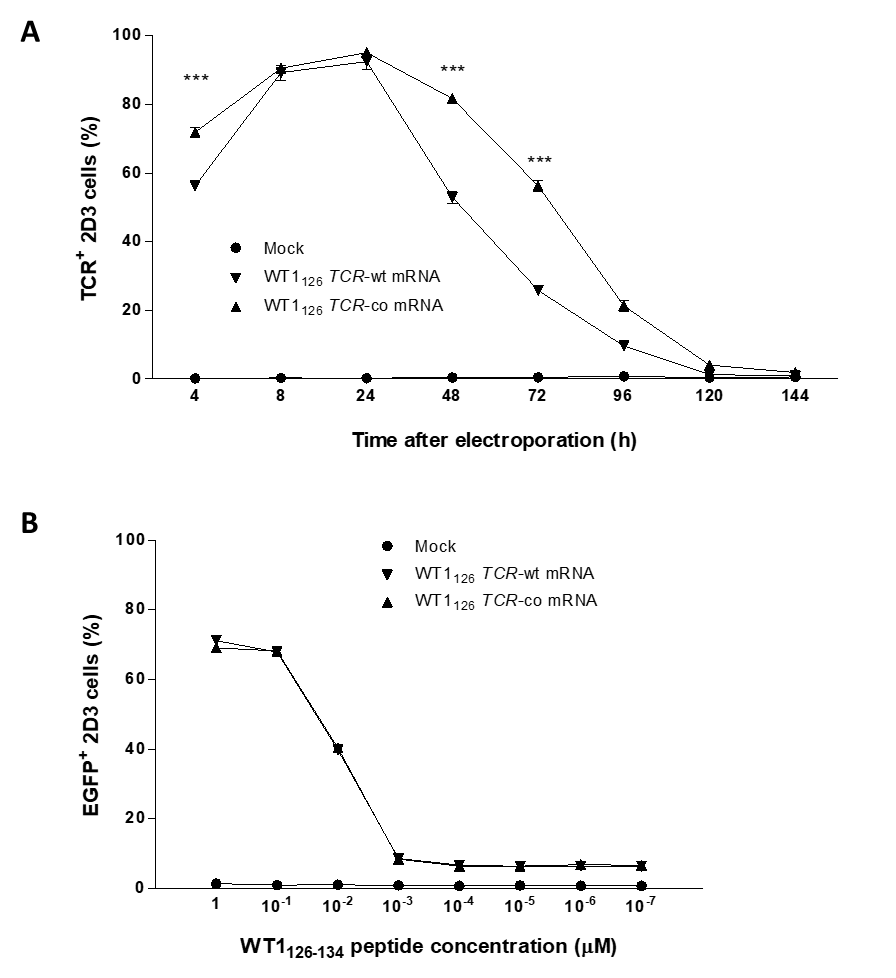
**Figure S1**

**Supplementary Figure 1 | Validation of WT1_126_ *TCR* mRNA in 2D3 cells.**

(**A**) Percentage over time of transgenic TCR expression after electroporation of TCRαβ-deficient 2D3 cells, as measured by anti-pan TCRαβ mAb staining (3 replicates, mean ± SEM). (**B**) Avidity of WT1_126_ TCR was assessed by TCR activation-mediated EGFP expression in 2D3 cells after co‑culture with WT1_126-134_ peptide-pulsed T2 cells. ****P* < 0.001; Mock, mock electroporation; WT1, Wilms’ tumor 1; wt, wild-type; co, codon-optimized.

**Figure S2**

**Supplementary Figure 2 | Specific downregulation of *TRAC* and *TRBC* transcripts 48h after DsiRNA electroporation.**

RT-qPCR was used to determine the levels of *TRAC* and *TRBC* transcripts in TCR^+^ Jurkat E6-1 cells 48h after DsiRNA/mock double sequential electroporation. The cells were electroporated first with TCR-specific or EGFP-specific DsiRNA or mock electroporated; 24h later they all underwent a second mock electroporation. Expression levels were normalized to the reference genes importin-8 and ribosomal protein L13A and analyzed relative to mock/mock double sequential electroporation. *TRAC*, T-cell receptor alpha constant region; *TRBC*, T-cell receptor beta constant region; Mock + Mock, double sequential mock electroporation; DsiRNA_EGFP_ + Mock, electroporation with Dicer-substrate small interfering RNA directed against *EGFP* mRNA followed by mock electroporation; DsiRNA + Mock, electroporation with Dicer-substrate small interfering RNAs directed against *TRAC* and *TRBC* followed by mock electroporation.

**Figure S3**

**
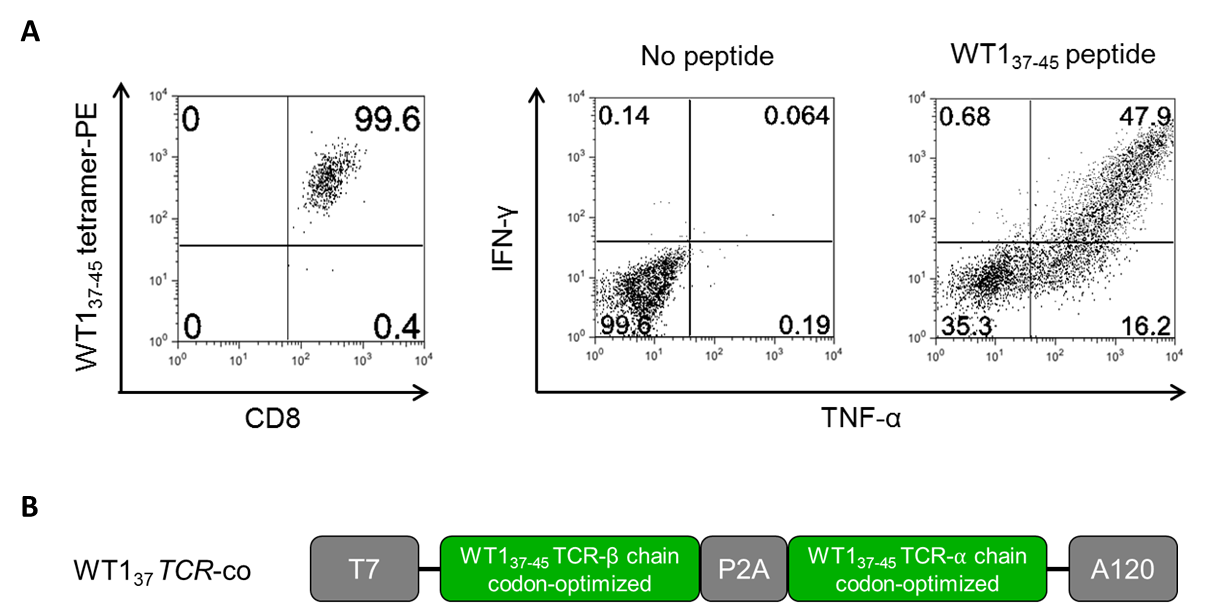
**

**Supplementary Figure 3 | Isolation and characterization of WT1_37-45_-specific CTL clone.**

(**A**) WT1_37-45_/HLA-A*02:01 tetramer staining and WT1_37-45_ peptide-specific IFN-γ and TNF-α production of the WT1_37-45_-reactive CTL clone. The percentage of cells is indicated in each quadrant. (**B**) Schematic representation of pST1 plasmid vector containing the WT1_37-45_-specific codon-optimized (WT1_37_ *TCR*-co) TCR cassette. WT1, Wilms’ tumor 1; wt, wild-type; co, codon‑optimized; T7, T7 promoter; P2A, picornaviral 2A-like sequence; A120, 120-mer poly-A tail.
